# *In vitro*-*in vivo* discord: A preclinical study of AZD2716 and its racemate with comparison to varespladib for the development of snake venom sPLA2 inhibitors

**DOI:** 10.1016/j.toxcx.2026.100243

**Published:** 2026-02-12

**Authors:** James L. Hearth, Yulia Surovtseva, Zuzana Karjala, Nicholas R. Casewell, Kate Giang, Matthew R. Lewin

**Affiliations:** a University of San Francisco, San Francisco, CA, USA; b Yale Center for Molecular Discovery, Yale University, New Haven, CT, USA; c Pacific BioLabs, Hercules, CA, USA; d Centre for Snakebite Research & Interventions, Liverpool School of Tropical Medicine, Liverpool, UK; e Carnegie Mellon University, Pittsburgh, PA, USA; f Ophirex, Inc., Corte Madera, CA, USA; g California Academy of Sciences, San Francisco, CA, USA

**Keywords:** sPLA2, Inhibitor, Snakebite, Envenoming, Small-molecule

## Abstract

We evaluated a family of repurposed sPLA2 inhibitors as novel candidate snakebite envenoming therapeutics. Stereospecific (*R*)-7 AZD2716 and its racemic mixture were compared to varespladib in an *in vitro* sPLA2 assay against a sample of 26 venoms from medically important snake species from five continents. All compounds demonstrated potent nano-to picomolar IC_50_ values, comparable to the benchmark inhibitory profile of varespladib. Surprisingly, however, this *in vitro* efficacy did not translate to survival in an *in vivo* mouse model under GLP standard conditions at an independent third party laboratory. In animal rescue studies evaluating both oral and IV dosing against the same four high sPLA2 venoms, varespladib demonstrated more consistent survival duration versus the chirally separated AZD2716 enantiomer and racemate following single-dose intravenous or two-dose oral drug administration. Additionally, the stereospecific AZD2716 did not provide the same survival advantage as the racemic mixture and neither molecule resulted in the same survival advantage as varespladib *in vivo* (p < 0.05), despite similar *in vitro* potency. These findings highlight the importance of following *in vitro* inhibition assays with preclinical studies in drug candidate selection for lead compounds and advancement to clinical development.

## Highlights

• This work identifies a new family of potentially repurposed sPLA2 inhibitors with potent *in vitro* inhibition across a diverse cohort of snake venoms.
• Surprisingly, these inhibitors showed inconsistent *in vivo* efficacy in a mouse model conducted under GLP reporting standards by an independent, third party, contract laboratory.
• These differences highlight the importance of preclinical testing as *in vitro* potency may not translate to *in vivo* efficacy.

## Graphical abstract

**Figure:**
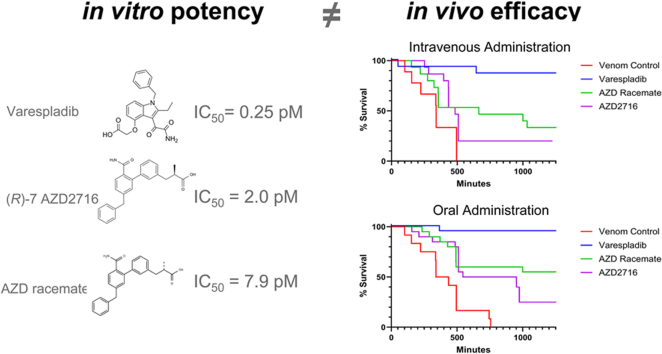

**Key Contribution:** This work identifies a new family of repurposed sPLA2 inhibitors with potent *in vitro* inhibition across a diverse cohort of snake venoms but inconsistent *in vivo* efficacy in a mouse model conducted under GLP reporting standards by an independent, third party, contract laboratory. These differences highlight the importance of preclinical testing as *in vitro* potency may not translate to *in vivo* efficacy. Nevertheless, the addition of new sPLA2 inhibitors to the toolkit of researchers may yield yet to be recognized benefits.

## 1 Introduction

Over 5 million people are bitten by snakes annually, of whom an estimated 138 000 die (Williams et al., 2019). Reliance on intravenous treatment, as is the case for currently available antibody-based antivenoms, generally limits the applicability of these snakebite therapeutics to more equipped settings and trained clinical personnel, and it is estimated that 75% of snakebite deaths occur prior to the patient reaching definitive care (Mohapatra et al., 2011) such that a “time-of-bite” treatment remains an unmet need. Recently, animal studies of several small-molecule therapeutics (SMTs) have highlighted their promise in addressing the initial, out-of-hospital treatment of snakebite envenoming, with the potential to reduce time to treatment — a major potential advantage over existing antibody-based antivenom therapy (Laustsen et al., 2018). SMTs may address this gap with their potent inhibition of some important and broadly conserved venom components, shelf stability, lower production costs, and ease of administration including via the oral route (Bulfone et al., 2018).

Of the druggable snake venom targets, the secretory phospholipase A2 (sPLA2) class is among the most compelling because of its multifunctional role, high toxicity, and ubiquity being found in varying proportions in upwards of 95% of venomous snake species (Tasoulis and Isbister, 2017). Snake venom sPLA2s are “multifunctional” toxins able to induce a wide variety of toxic signs and symptoms including neurotoxicity, hemolysis, coagulopathy, cytotoxicity, and severe pain (Gutiérrez and Lomonte, 2013). Recently, varespladib, a repurposed SMT that potently and broadly inhibits snake venom PLA2s, was shown to prevent or delay lethality in murine and porcine rescue models of neurotoxic and hemo/cytotoxic envenoming, demonstrating strong potential for introduction as an adjunct snakebite therapy (Lewin et al., 2018a; Zinenko et al., 2020; Tan et al., 2022; Gilliam et al., 2023). Varespladib and its orally bioavailable prodrug, varespladib-methyl, have recently concluded Phase II clinical trials in the United States and India (Gerardo et al., 2024). At this time, there are no approved sPLA2 inhibitors for human use.

Varespladib and a counterpart family of AstraZeneca sPLA2 inhibiting compounds were being developed contemporaneously for treatment of cardiovascular disease (Nicholls et al., 2014; Giordanetto et al., 2016). These compounds were reported to have similar potency to varespladib but were abandoned following the early termination of the varespladib VISTA-16 trial, due to futility relating to an interim analysis and a question of safety with long-term use (Nicholls et al., 2014). Several of the reported characteristics of the AZD compounds were of interest to us: the AZD investigators suggested that stability and oral permeability of AZD2716 (IUPAC (2R)-3-[3-(5-benzyl-2-carbamoylphenyl)phenyl]-2-methylpropanoic acid) could offer several advantages over other sPLA2 inhibitors such as varespladib (Giordanetto et al., 2016). AZD2716 in its native form had a reported oral bioavailability of over 80% in rats and dogs (Giordanetto et al., 2016) compared to ∼48% for varespladib in their studies. Bioavailability is an important consideration in the development of an optimized “time-of-bite” oral SMTs. We reasoned that the high oral bioavailability of AZD2716 could potentially enable more rapid field administration, possibly lowering the costs of manufacturing and improve the overall management of snakebite envenoming compared to varespladib.

To begin understanding how two drugs with similar potency might behave in the setting of envenoming, we investigated the *in vitro* inhibitory potential of AZD2716 in both racemic and stereoisomer (*R*)-7 forms, as well as its efficacy in an *in vivo* murine delayed-administration rescue study of 4 different snake venoms. We aimed to compare AZD2716 to varespladib in experimental envenoming models where varespladib has been previously shown to improve survival in animal models (Lewin et al., 2016; Peggion and Tonello, 2021, Tan et al., 2022). These studies were conducted at a contract research laboratory under GLP conditions with evaluation of sPLA2 inhibitors reported similarly potent to varespladib and with favorable formulation and pharmacokinetic (PK) characteristics (Giordanetto et al., 2016).

## 2 Materials and methods

### 2.1 *In vitro* experiments

The methods for *in vitro* experiments has been described in our previous work (Lewin et al., 2016). Briefly, however: Experiments were performed to assess sPLA2 activity *in vitro* using 1,2-bis(heptanoyl) glycerophosphocholine as a substrate. The Bee Venom PLA2 Control was a 100 μg/mL solution of bee venom PLA2 supplied as a positive control from commercially acquired kits (Abcam kit catalog number ab133089). Assay optimization, screening and dose response measurements were performed at the Yale Center for Molecular Discovery. Experiments were performed in an assay buffer containing 25 mM Tris-HCl, pH 7.5 (Cayman Chemical, Ann Arbor, MI, USA), 10 mM CaCl_2_ (J. T. Baker), 100 mM KCl (Sigma, St. Louis, MO, USA), 0.3% Triton X-100 (Fluka) and 454 μM DTNB (5,5-dithio-bis-(2-nitrobenzoic acid) (Cayman Chemical) in clear, non-treated 384-well plates (Corning, NY, USA). Venoms (Miami Serpentarium, Punta Gorda, FL, USA, Kentucky Reptile Zoo, Slade, KY, USA, and Sigma) were reconstituted in 1 × phosphate-buffered saline (Lonza, Basel, Switzerland) to a concentration of 10 000 μg/mL. Crude, unfractionated lyophilized venom purchased from Sigma (*Echis carinatus* and *Daboia russelii*) or the Miami Serpentarium (all others) was used in all cases. Varespladib HCl (free acid) was purchased from Chemietek (Indianapolis, IN, USA) and dissolved in DMSO. AZD2716 and related compounds were custom synthesized by WuXi Apptec (Shanghai, China). Instrumentation used included Echo acoustic dispenser (Beckman), Thermo Multidrop liquid dispensers (Hudson, NH, USA), and Tecan infinite M1000 platereader (Männedorf, Switzerland).

The activity of venoms with 0.375 mM 1,2-bis(heptanoyl) glycerophosphocholine (Cayman Chemical), the sPLA2 substrate, was selected based on kinetic enzymatic assays conducted at room temperature. Concentrations of venom were selected for screening and potency studies in which high sPLA2 activity was observed relative to any background activity of no venom control wells, and for which there was negligible substrate depletion at 60 min. The range in final concentrations for venoms used in assays was 0.0037–5 μg/mL, demonstrating large differences among venoms in the proportion or relative sPLA2 activity for this substrate.

For inhibitor and dose-response testing, 10 μL of snake venom or bee venom (positive control) was added to assay plates using a multichannel pipetman (Matrix, Hudson, NH, USA) or a multidrop dispenser (Thermo, Hudson, NH, USA). Compounds from the prepared serial dilution master plates dissolved in DMSO were added to assay plates using an Echo acoustic dispenser (Beckman) to transfer 20 nL of compounds. Final DMSO concentrations in the assay were 0.1%. Substrate was then added in 10 μL for a final assay volume of 20 μL. Control populations were included on each plate in replicate wells. The negative control wells were vehicle (DMSO-only) with no small molecule compound. The positive control to simulate full venom activity inhibition were wells in which no venom was added, and assay buffer added in its place. Assay signals were measured at initiation and after 60 min of reaction time at room temperature. Signals were quantified on a Tecan infiniTe M1000 plate reader measuring absorbance at 405 nm. Signals at initiation were subtracted from the signals at 60 min. These background-corrected values were normalized to the mean of replicate negative and positive control wells within the plate. To define the normalization scale, the mean of the negative control well signals, representing full venom activity, was normalized to 100% effect and the mean of positive control well signals, representing complete inhibition of venom activity, was normalized to 0% effect and test wells within the plate were scaled accordingly. These calculations were performed in Microsoft Excel. Data were transferred to GraphPad Prism (6th edition, 2014, La Jolla, CA, USA) plotted and fit to models, such that *p*IC_50_ are reported as described below. Tests of significance between compounds were calculated by 2-tailed Student's *t*.

### 2.2 *In vivo* experiments

Animal work was approved by the Pacific BioLabs (Hercules, California) Institutional Animal Care and Use Committee and the work was performed under Good Laboratory Practices on site**.** The California Academy of Sciences was not involved in any of the animal work.

Groups of CD-1 male mice with body weights ranging from 24.8 to 35 g were exposed to venom from four different venomous snake species: Eastern coral snake (*Micrurus fulvius,* 0.5 and 1.0 mg/kg), coastal taipan (*Oxyuranus scutellatus,* 0.1875 mg/kg), Mojave rattlesnake (*Crotalus scutulatus,* 1.0 mg/kg*)*, and Russell's viper (*Daboia russelii*, 1.0 mg/kg). Venoms were received as powders and diluted to final concentrations with 1X Phosphate Buffered Saline (PBS). Mice were envenomed via subcutaneous injection in the scruff (*M. fulvius, O. scutellatus, C. scutulatus*) or intraperitoneally at a volume of 10 mL/kg *(D. russelii*). Venom challenge routes were selected based on established envenoming models (Lewin et al., 2018a; Hasan et al., 2025). The lethality of each snake venom was assessed in three control animals. *M. fulvius* venom was assessed at both 0.5 and 1.0 mg/kg; however, clinical signs were very mild in animals receiving 0.5 mg/kg and we determined that a dosage of 1.0 mg/kg was more suitable for this study. Experimental groups received treatment (n = 5) within 10 min of experimental envenoming. Venom doses were based on historical data from these same venom lots used in prior studies at the same facility with the same strains and weights of mice. Three drug treatments were also evaluated in this study in groups of 5 experimental animals: AZD2716 ((*R*)-7 enantiomer), Racemate, and Varespladib HCl (LY315920). Racemate and AZD2716 were administered either intravenously (i.v.) via tail vein at dose volume 5 mL/kg (5 mg/kg) or orally (p.o.) via oral gavage at dose volume 10 mL/kg (10 mg/kg). Varespladib (LY315920) HCl (5 mg/kg, dissolved in bicarbonate/dextrose) was administered via tail vein at dose volume 5 mL/kg and varespladib-methyl (LY333013) was administered orally by gavage at dose volume 10 mL/kg (10 mg/kg). Animals received treatments within 10 min following venom dosing in the intravenous administration groups and within 10 min and at 6 h in the oral administration groups.

After envenoming and/or treatment, animals were monitored continuously for approximately 8 h and then on an hourly basis thereafter except overnight. Animals were evaluated for clinical signs of toxicity including but not limited to: abnormal posture; appearance of skin, fur, eyes, and mucous membranes; urine and fecal output; and changes in locomotor behavior or respiration. Mice were evaluated for paralysis, prolonged or severe respiratory distress, and/or moribundity. Once these clinical signs were present, the animals were euthanized. Close monitoring of animals continued until the conclusion of the study (Clinical observations in Supplementary Data).

### 2.3 Statistical methods

***In vitro:*** IC_50_ data were compared to varespladib after log-transformation to pIC_50_ (-log_10_(IC_50_)) to normalize variance and approximate a normal distribution. IC_50_ data are typically log-normal and span multiple orders of magnitude. For example, a venom with IC_50_ of 2.5 x 10^−13^ M (0.25 pM) is transformed to a pIC_50_ of 12.6 for reporting and statistical analysis (Srinivasan and Lloyd, 2024) Comparisons to varespladib were performed using paired, two-tailed Student's *t*-tests. Comparison of pIC_50_ values between elapid and viperid venoms were assessed with linear models with species-clustered standard errors to account for repeated measurements across compounds.

***In vivo:*** Log-rank and Holm-Sidak multiple comparisons testing were used to evaluate survival differences between aggregated cohorts following Kaplan-Meier analysis. Restricted Mean Survival Time (RMST) data and CI/p-values were estimated using nonparametric bootstrapping through 2000 iterations. This method was chosen due to non-normal, censored survival data, providing uncertainty estimates without the assumption of proportional hazards or parametric survival distributions.

## 3 Results

### 3.1 *In vitro* inhibition assay

We first tested the effects of AZD2716, its racemate (“Compound 7” (Giordanetto et al., 2016)), and varespladib using *in vitro* sPLA2 activity assays which utilize snake venom as sPLA2 source (or bee venom as a PLA2 control) and 1,2-bis(heptanoyl) glycerophosphocholine as a substrate. Upon hydrolysis of the substrate by PLA2, free thiols are detected using DTNB (5,5-dithio-bis-(2-nitrobenzoic acid)), with dose-dependent signal reduction in the presence of small molecule sPLA2 inhibitors. Concentrations of venom were selected for screening and potency studies in which high sPLA2 activity was observed relative to any background activity of no venom control wells, and for which there was negligible substrate depletion at 60 min (Supplementary Fig. 3). Using this *in vitro* assay, we showed that AZD2716 and racemate were comparably potent to varespladib with IC_50_ concentrations in the low nano-to pico-molar range (Supplementary Fig. 2, and Table 1). When all inhibitors were compared against the same panel of venoms in the same dose-response-curve runs, the average pIC_50_ of varespladib was 12.6 ± 1.2, AZD2716 11.7 ± 0.9, and AZD racemate 11.1 ± 0.9 (Table 1), corresponding to IC_50_ values of 2.5 x 10^−13^ M (1.6 x 10^−14^ to 4.0 x 10^−12^) for varespladib, 2.0 x 10^−12^ M (2.5 x 10^−13^ to 1.6 x 10^−11^) for AZD2716, and 7.9x10^−12^ M (1.0 x 10^−12^ to 6.3 x 10 ^−11^) for AZD racemate.

**Table 1:** pIC_50_ [-log(IC_50_) ] by Snake Venom by Continent of species origin. On average, varespladib was more potent in the *in vitro* assays compared to either AZD2716 or its racemic mixture (p < 0.001). All dose response graphs are shown in Supplementary Figure (2). All samples were run in duplicate. (∗) denotes different venom lots or sourcing for venoms from the same species.

Continent	Snake Species	pIC_50_ (-log(IC_50_)) and Standard Error of the Mean (SEM)					
		Varespladib	SEM	AZD2716	SEM	AZD Racemate	SEM
North America	*Agkistrodon bilineatus*	12.7	0.02	12	0.04	11.6	0.04
North America	*Agkistrodon contortrix*	13.2	0.02	12.4	0.05	12	0.04
North America	*Agkistrodon contortrix*∗	13	0.02	12.3	0.05	11.9	0.04
North America	*Agkistrodon conanti*	12.7	0.03	12.1	0.05	11.7	0.04
North America	*Agkistrodon conanti* ∗	12.6	0.02	11.8	0.04	11.5	0.03
North America	*Agkistrodon piscivorus*	12.9	0.02	12.2	0.05	11.8	0.04
South America	*Bothrops atrox*	13.4	0.02	12.4	0.06	11.8	0.05
South America	*Bothrops moojeni*	12.7	0.02	11.5	0.05	11.1	0.05
North America	*Crotalus adamanteus*	13.4	0.03	12.2	0.05	11.8	0.05
North America	*Crotalus atrox*	13	0.03	12	0.05	11.6	0.05
South America	*Crotalus durissus durissus*	11.3	0.04	10.7	0.06	10.2	0.05
South America	*Crotalus durissus terrificus*	11.4	0.04	10.7	0.06	10.2	0.05
North America	*Crotalus viridis*	12.9	0.02	11.8	0.05	11.4	0.05
North America	*Crotalus viridis* ∗	12.9	0.03	11.8	0.05	11.4	0.05
North America	*Crotalus scutulatus scutulatus*	11.6	0.03	10.9	0.05	10.4	0.04
Africa	*Vipera ammodytes*	13.6	0.03	13.3	0.04	12.6	0.04
Africa	*Naja melanoleuca*	12	0.04	11.3	0.03	10.6	0.04
Africa	*Naja haje*	11.7	0.03	11	0.03	10.4	0.04
Africa	*Naja mossambica*	11	0.04	10.6	0.04	9.9	0.05
Africa	*Naja melanoleuca ∗*	11.6	0.03	11.1	0.04	10.6	0.04
Africa	*Bitis arietans*	10.9	0.05	11.5	0.03	10.9	0.03
Africa	*Bitis arietans* ∗	12.4	0.03	11.3	0.03	10.7	0.03
Africa	*Dispholidus typus*	11.6	0.02	11.6	0.02	10.9	0.03
Africa	*Echis ocellatus*	9.8	0.04	8.4	0.05	7.8	0.07
Africa	*Echis pyramidium*	10.7	0.04	11.3	0.04	10.7	0.04
India	*Bungarus caeruleus*	14.5	0.04	11.9	0.05	11.5	0.05
India	*Bungarus fasciatus*	14.2	0.05	11.8	0.06	11.4	0.05
India	*Naja naja*	13.4	0.02	10.9	0.05	10.5	0.05
India	*Daboia russelii*	14.8	0.03	12.8	0.05	12.3	0.05
India	*Echis carinatus sochureki*	14.8	0.03	13	0.06	12.5	0.06
India	*Echis carinatus*	14.8	0.03	13.1	0.06	12.6	0.05
Oceania	*Oxyuranus scutellatus*	12.9	0.03	11.5	0.02	10.9	0.03
Oceania	*Oxyuranus scutellatus* ∗	12.4	0.04	11.4	0.02	10.7	0.03
Oceania	*Oxyuranus scutellatus* ∗	12.6	0.03	11.6	0.02	10.9	0.03
Oceania	*Pseudechis australis*	10.6	0.04	11.7	0.03	11	0.03
**Average**		**12.6** ± **1.2**		**11.7** ± **0.9**		**11.1** ± **0.9**	

#### 3.1.1 Comparison of pIC_50_ values between viperid and elapid venoms

Across the venoms tested, viperid venoms trended toward higher pIC_50_ values (greater inhibition) than elapid venoms for both AZD2716 and its racemate. No difference was detected for varespladib (p = 0.60). Using a linear model with species-clustered standard errors estimated a viperid-elapid difference of 0.473 log units for AZD2716 and 0.553 for racemate, though these values did not reach statistical significance after Holm adjustment (p = 0.13, p = 0.11 respectively).

### 3.2 *In vivo* mouse studies

We tested the effects of AZD2716, its racemate, and varespladib treatment on acute venom-induced toxicity following experimental administration of venoms from the four different species of snake for which we could make direct comparisons between studies using the same venom and drug lots—*M. fulvius* (Eastern coral snake), *O. scutellatus* (Coastal Taipan), *C. scutulatus* (Mojave Rattlesnake), and *D. russelii* (Russell's viper). Studies were conducted on two dates a week apart, both under GLP standard conditions.

**Fig. 1:**
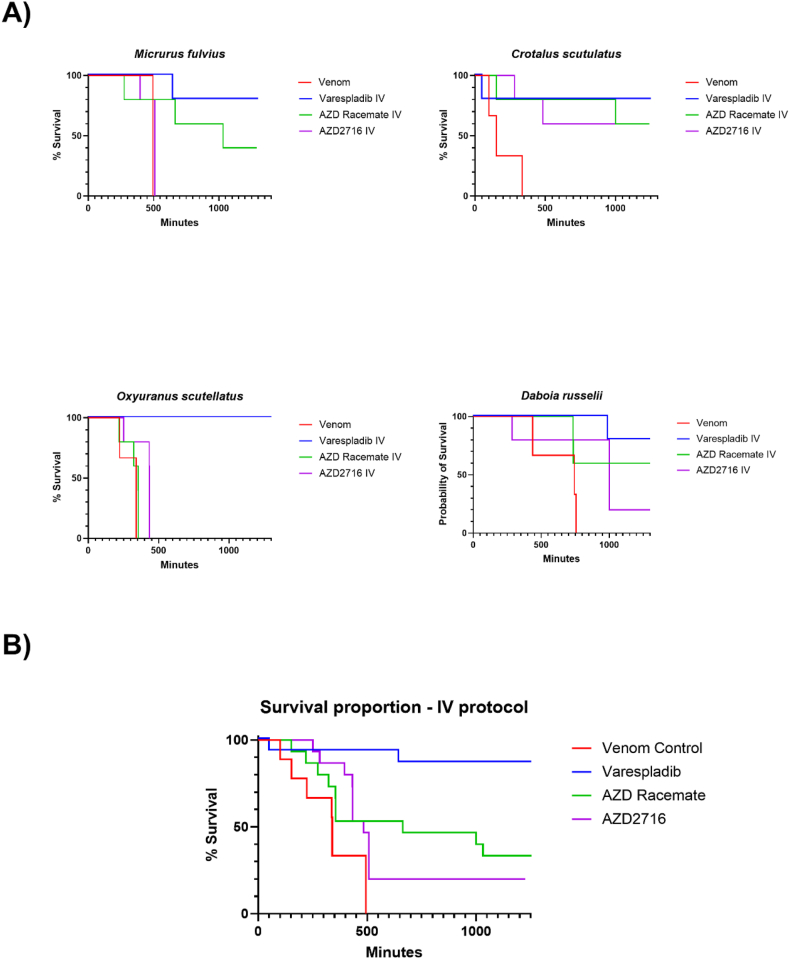
Survival of mice following experimental envenoming and intravenous (IV) rescue protocol with varespladib, AZD2716, or AZD racemate. **A)** Control animals received venom only (n = 3) based on historical data from these venom lots. Experimental groups received treatment (n = 5) within 10 min of experimental envenoming. Venom doses were based on historical data from these same venom lots used in prior studies at the same facility. **B)** Combined survival of mice following experimental envenoming and IV rescue protocol with varespladib, AZD racemate, and AZD2716 (Control n = 12, Treatment n = 20). In aggregate, varespladib-treated animals survived longer than its non-substituted indole counterparts (p < 0.05).

Briefly, mice were exposed to venom either subcutaneously (*M. fulvius, O. scutellatus, and C. scutulatus*) or intraperitoneally (*D. russelii*) and then received either (1) no treatment (control group; n = 3), (2) intravenous AZD2716 (5 mg/kg IV), (3) oral AZD2716 (10 mg/kg PO), (4) intravenous AZD2716 racemate (5 mg/kg IV), (5) oral AZD2716 racemate (10 mg/kg PO), (6) intravenous varespladib HCl (LY315920, 5 mg/kg IV) or (7) oral varespladib-methyl (10 mg/kg PO). All experimental groups receiving drug were in groups of n = 5 animals. Survival results for intravenously (IV) treated animals are shown in Fig. 1, immediately below.

**Fig. 2:**
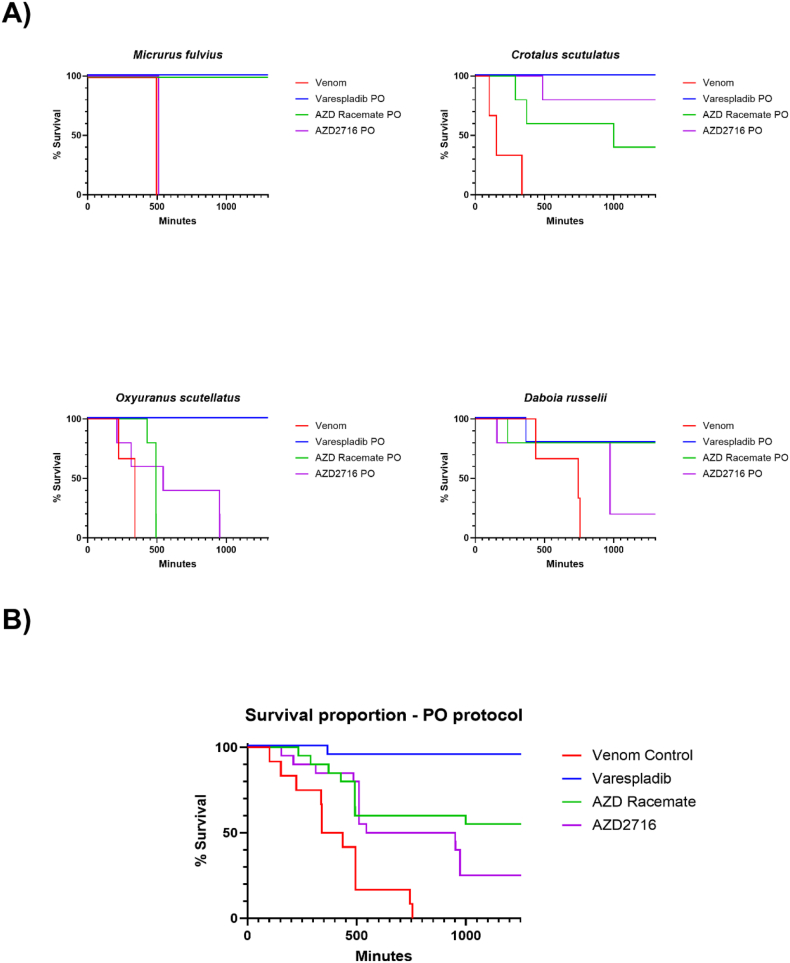
Survival of mice following experimental envenoming and oral (PO) rescue protocol with varespladib-methyl, AZD2716, or racemate. **A)** Control animals received venom only (n = 3) based on historical data from these venom lots. Experimental groups received treatment (n = 5) within 10 min of experimental envenoming. Venom doses were based on historical data from these same venom lots used in prior studies at the same facility with the same strains and weights of mice. **B)** Combined survival of mice following experimental envenoming and PO rescue protocol with varespladib-methyl, AZD racemate, and AZD2716 (Control n = 12, Treatment n = 20). In aggregate, varespladib-methyl-treated animals survived longer than its non-substituted indole counterparts (p < 0.05).

For both IV (Fig. 1 A) and PO (Fig. 2 A) treatment groups, across all species tested, varespladib demonstrated the greatest benefit to survival, with comparably improved survival results between AZD2716 and its racemate. However, none of the drug treatments had statistically significant differences in survival between each other as determined through Kaplan-Meier survival analysis when examined at the species level, possibly because of the small group sizes.

Overall treatment effect aggregating survival data across all species was analyzed in a species-blind manner for both IV and PO rescue protocols (Fig. 1 B & 2 B). For both administration routes, venom-only control animals (n = 12 per protocol) exhibited rapid and complete mortality within the observation window. All compounds tested significantly improved survival versus the venom control groups (X^2^ = 26.1, p < 0.01). Varespladib offered the strongest survival benefit (X^*2*^ = 20.28, p < 0.01) compared to AZD2716 (X^2^ = 5.93, p < 0.05) and its racemate (X^*2*^ = 5.31, p < 0.05) when tested with Holm-Sidak multiple comparisons testing. In contrast, no statistically significant difference was detected for either administration route between AZD2716 and its racemate (p > 0.1).

### 3.3 Restricted mean survival time (RMST) analysis

**Fig. 3:**
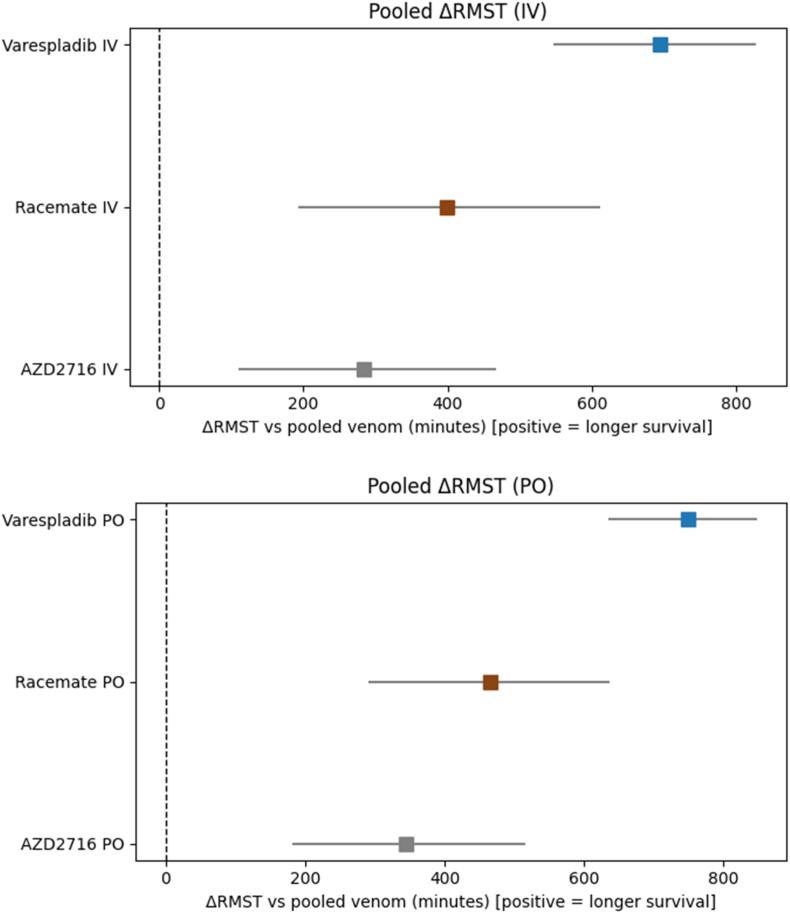
Change in Restricted Mean Survival Time (ΔRMST) for experimentally envenomed mice treatment by IV (top) or PO (bottom) protocols with varespladib, AZD2716, and AZD racemate. ΔRMST represents the difference in restricted mean survival time relative to pooled venom-only controls. Cutoff (τ) 1200 min, 2000 bootstraps.

To better characterize treatment effects in the small cohorts where several treatment groups did not reach median survival, Restricted Mean Survival Time (RMST) was analyzed which reflects the average survival time within a fixed observation period and provides an intuitive measure of a treatment's mortality delay. RMST was used to compare magnitude and direction of survival benefits across compounds and venom families by route of drug administration (Fig. 3).

#### 3.3.1 RMST results from intravenous administration

Varespladib produced the largest improvement in RMST, with an average ΔRMST of 694 min (+746 in elapids, +642 in vipers). Compared to varespladib, the AZD racemate offered a shorter survival benefit overall, albeit with a greater divergence between efficacy versus venoms from different families (ΔRMST vs. elapids: +217 min, ΔRMST vs. vipers +581 min). AZD2716 had a negligible or minimal effect in the elapid model (ΔRMST +44 min) but provided a more clear benefit versus viper venoms (+524 min), comparable to its racemate and varespladib (for detail see Supplementary Fig. 1).

#### 3.3.2 RMST results from oral administration

Varespladib conferred the greatest survival advantage, with no deaths in the elapid-envenomed animals receiving varespladib and one death in 10 of the viper venom group of animals having a ΔRMST of +695 min. Although AZD2716 was unexpectedly ineffective against elapid venoms (ΔRMST +155 min), it was noticeably more effective against viperid venoms tested (ΔRMST +535 min) compared to its racemate with an RMST of 442 min in elapids and 488 min in vipers.

### 3.4 *In vivo* survival results by venom type

*M. fulvius* envenoming: Control mice receiving *M. fulvius* (eastern coral snake) venom (n = 3) met euthanization criteria within 8 h (RMST = 472 min). Neither route of administration for AZD2716 nor its racemate significantly improved survival of the animals in this group (AZD2716 IV ΔRMST = −8 min [–53 to 14], PO ΔRMST = 16 min [15 to 16], racemate IV ΔRMST = 293 [95% CI 69 – 505]). Four of five mice treated with varespladib IV survived (ΔRMST = 457 min [314 to 577]) and all mice treated with varespladib PO survived. Notably, all animals that received the racemate orally survived to the end of the study. Racemate-treated animals exhibited slight clinical signs such as piloerection, but were otherwise bright, alert, and responsive, similar to the varespladib group.

*O. scutellatus* envenoming: Control mice administered venom from *O. scutellatus* (coastal taipan) (n = 3) met euthanization criteria with an RMST of 304 min. IV AZD2716 afforded limited protection (ΔRMST = 96 min [20 to 174]), and the oral route had slighter higher efficacy (PO ΔRMST = 293 min [18 to 562]). The racemate offered negligible improvement (IV ΔRMST = 21 min [−46 to 99], PO ΔRMST = 176 [126 to 244]). In contrast, all animals receiving varespladib survived through the study period. Animals receiving IV varespladib had improved clinical signs but by the end of the study all animals displayed a scruffy coat suggesting potential relapse due to wearing off of the drug effect and comparably long half-life of the venom, but no animals in the AZD treatment groups survived to the end of the study.

*C. scutulatus* envenoming: Control mice receiving *Crotalus scutulatus* (Mojave rattlesnake) venom (n = 3) met euthanasia criteria with an average RMST of 197 min. All compounds markedly improved survival. Among parenterally administered compounds, AZD2716 racemate and varespladib were comparable (ΔRMST = 633 min [95% CI 233 – 882] and 613 min [95% CI 250 – 882], respectively). AZD2716 improved RMST by 556 min [95% CI 231 – 847]. All animals receiving oral varespladib survived until the end of the study period. Orally administered AZD2716 and racemate both improved survival (ΔRMST = 700 min [95% CI 457 – 882] and 535 min [95% CI 233 – 820]), but there was at least one death in each group (2 to 3 deaths out of five mice in each instance. Varespladib mice were bright, alert, and responsive except for one hypoactive animal with ptosis after 6 h, resolving within an hour of the 6-h mark second dose.

*D. russelii* envenoming: Control mice receiving venom from *D. russelii* (Russell's viper) (n = 3) exhibited an RMST of 645 min. All compounds appear to extend survival. Among IV-administered drugs, varespladib had the greatest improvement in survival (ΔRMST = 352 min [95% CI 241 – 561]). AZD2716's racemate and AZD2716 both improved survival to a lesser extent (ΔRMST = 249 min [95% CI 83 – 461] and 212 min [95% CI -69 – 461], respectively). Oral administration yielded lesser, albeit more closely comparable results, with varespladib extending survival by 228 min [95% CI -30 – 461], racemate by 202 min [95% CI -152 – 461.3], and AZD2716 by 170 min [95% CI -162 – 446]. Clinical signs including lethargy, tremors, and ptosis were noted in all animals within 6 h of venom administration. Surviving mice were bright, alert, and responsive until the end of the study.

## 4 Discussion

We examined the *in vitro* inhibitory potential of AZD2716 and its racemate against reconstituted whole venoms from five continents to compare the *in vitro* potency of sPLA2 inhibition and their effects on survival in an *in vivo* mouse model. This work introduces a second family of repurposed, highly potent synthetic sPLA2 inhibitors to the field of toxinology in comparison with previously characterized varespladib (Lewin et al., 2016; Knerr et al., 2018). AstraZeneca abandoned development of AZD2716 and its racemate following publication of the VISTA-16 varespladib trial prior to human clinical testing for long-term use (16 weeks) in patients with high risk acute coronary syndromes and which was terminated early because of a possible injury signal (Nicholls et al., 2014). The 7-day varespladib dosing protocol used in the BRAVO trial did not show a cardiac signal but patients with known coronary disease were excluded (Gerardo et al., 2024). Giordanetto and colleagues described *in vitro* potency of AZD2716 and claimed superior bioavailability reported in animal models (Giordanetto et al., 2016). While our study did not examine or compare these attributes, despite high potency observed *in vitro*, administration of these non-indole sPLA2 inhibitors did not appear to translate to consistent survival advantage when compared to varespladib, a substituted indole, in envenoming studies performed under GLP conditions. We did not specifically examine this, but there might be differences in how varespladib and AZD2716 interact with Group I sPLA2s *in vivo*. As well, Giordanetto was focused on mammalian sPLA2s and our study focuses on ophidian sPLA2s. Overall, the *in vivo* results were surprising because while there were slight differences in in vitro potency, bioavailability of AZD compounds was reported to be superior to varespladib as reported by Giordanetto.

Differences in efficacy between *in vitro* and *in vivo* effects highlight important considerations in drug development. While AZD2716 (the R-enantiomer) has been shown by Giordanetto and our own study to be potent *in vitro* (Table 1), there did not appear to be a consistent effect on survival in these first animal studies (Fig. 1, Fig. 2, Fig. 3). There are several possible explanations including but not limited to *in vitro* exposure which is constant and not subject to the pharmacokinetic and pharmacodynamics of living organisms. We did not seek to examine the reason for the difference in survival, but some differences between the structures of varespladib and the AZD compounds could explain differences in *in vivo* efficacy despite comparable *in vitro* potency. For example, varespladib has an inflexible indole skeleton that sits in the hydrophobic groove of sPLA2 while the AZD compounds have significant degrees of freedom (Salvador et al., 2019; Barbosa et al., 2021, Salvador et al., 2021; Salvador et al., 2024; Salvador et al., 2026, in review). Considering the difference in efficacy after oral and IV treatment, different metabolism is another possible explanation for the discrepancy. This could also explain the different effects of the racemate and enantiomer as metabolism is often enantioselective (Coelho et al., 2021). Differing distributions or excretion may also play a role in the varied efficacy. Crystallographic and computational studies with venom toxins, such as those recently performed for varespladib in relation to *Bothrops* venoms, are underway and could be a useful approach to better understanding the structural bases for similarities and differences between these two highly potent inhibitors (Salvador et al., 2019, Barbosa et al., 2021; Salvador et al., 2026 [in review]).

AZD2716 has only a single chiral center and thus only two enantiomers. While Giordanetto et al. (2016) demonstrated that AZD2716 was the most potent of their series (Giordanetto et al., 2016) it is not clear if chirality is a factor in the lesser efficacy seen our animal models. Whereas varespladib has a well-established background of crystallographic study, the binding behavior of AZD2716 has yet to be examined. If drug candidates such as these were to be advanced, a developer might consider reducing cost of goods by developing the racemate for this indication rather than adding the more expensive chiral separation step to the development process. Other limitations include that this was only a single study and we did not test every compound in the AZD series reported by Giordanetto and colleagues or perform dose solution analyses for these compounds or varespladib. The AZD compounds were custom synthesized and while purified, were administered in the exact same excipient as reported by Giordanetto (Giordanetto et al., 2016). In addition, we only examined survival in our *in vivo* studies and did not examine sublethal effects such as correction or worsening of hematological abnormalities, preservation of kidney function or tissue injury. Despite these caveats, our study highlights several important considerations in drug development — most notably that apparent *in vitro* similarity between compounds might not translate to survival advantage in *in vivo* preclinical evaluation.

## CRediT authorship contribution statement

**James L. Hearth:** Writing – review & editing, Writing – original draft, Formal analysis, Data curation. **Yulia Surovtseva:** Methodology, Formal analysis, Data curation. **Zuzana Karjala:** Writing – review & editing, Methodology, Investigation. **Nicholas R. Casewell:** Validation, Investigation. **Kate Giang:** Formal analysis, Data curation. **Matthew R. Lewin:** Writing – review & editing, Writing – original draft, Visualization, Supervision, Software, Resources, Project administration, Methodology, Investigation, Funding acquisition, Conceptualization.

## Institutional review board statement

The study was conducted according to the study Protocol and applicable Pacific BioLabs Standard Operating Procedures on behalf of Ophirex, Inc. as the study sponsor and was not related to any work of the other participating institutions. Animal studies were performed outside the purview of the California Academy of Sciences.

## Funding

Ophirex, Inc. (internal research funds) and 10.13039/100014976Liverpool School of Tropical Medicine (10.13039/100010269Wellcome Trust/Internal research funds)

## Declaration of competing interest

MRL has stock in Ophirex, Inc. a Public Benefit Company and is no longer employed there (current address California Academy of Sciences, San Francisco, CA) JLH, YS, ZK, NC, and KG do not have any conflicts of interest to declare.

## Data Availability

Data will be made available on request.
