# Supplementary material for: In vitro-in vivo discord: A preclinical study of AZD2716 and its racemate with comparison to varespladib for the development of snake venom sPLA2 inhibitors
Source: Toxicon X. 2026 Feb 12;29:100243. doi: 10.1016/j.toxcx.2026.100243 (PMC12926600; doi:10.1016/j.toxcx.2026.100243)
Supplement: Multimedia component 1 [file mmc1.docx]

**Supplementary Materials:**

**Supplementary Table 1: IV – Restricted Mean Survival Time Results**

ΔRMST by Treatment and Family — IV

| Treatment | Elapid ΔRMST (min) | Elapid 95% CI | Elapid p_boot | Viper ΔRMST (min) | Viper 95% CI | Viper p_boot | Mean ΔRMST (min) |
| --- | --- | --- | --- | --- | --- | --- | --- |
| Varespladib | 746.4 | [625.5, 844.2] | 0.56 | 642.3 | [332.9, 865.2] | 0.53 | 694.4 |
| AZD Racemate | 216.7 | [-34.6, 492.2] | 0.48 | 580.9 | [307.9, 812.2] | 0.50 | 398.8 |
| AZD2716 | 43.9 | [-29.2, 120.9] | 0.53 | 524.1 | [215.3, 795.7] | 0.49 | 284.0 |

**Supplementary Table 2: PO – Restricted Mean Survival Time Results**

ΔRMST by Treatment and Family — PO

| Treatment | Elapid ΔRMST (min) | Elapid 95% CI | Elapid p_boot | Viper ΔRMST (min) | Viper 95% CI | Viper p_boot | Mean ΔRMST (min) |
| --- | --- | --- | --- | --- | --- | --- | --- |
| Varespladib | 802.0 | [743.8, 860.2] | 0.54 | 695.4 | [469.9, 887.0] | 0.52 | 748.7 |
| AZD Racemate | 441.7 | [206.6, 673.3] | 0.52 | 488.1 | [175.7, 760.2] | 0.50 | 464.9 |
| AZD2716 | 154.6 | [11.3, 315.6] | 0.49 | 535.2 | [267.2, 777.7] | 0.51 | 344.9 |

**Supplementary Figure 1: Restricted Mean Survival Time Results by Family (IV top, PO bottom)**


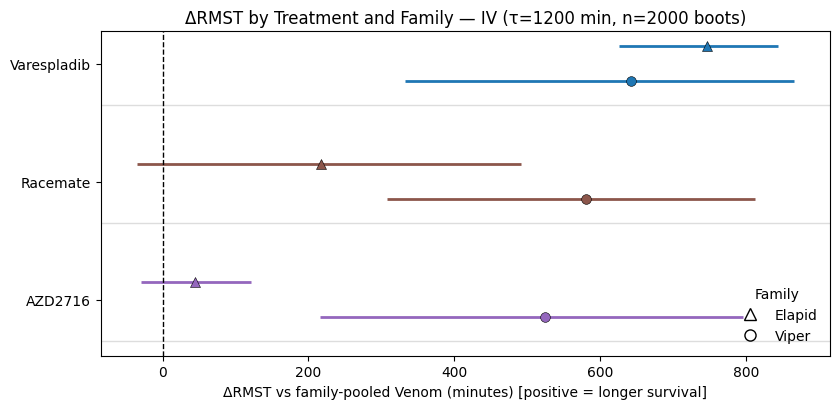


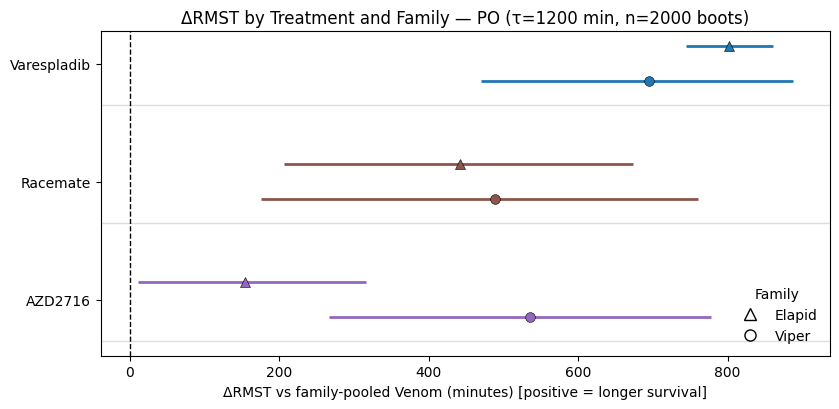


**Supplementary Figure 2: Dose-Response Curves for sPLA2 assay**

Dose-response curves for sPLA2 inhibition across 35 snake venoms for varespladib, AZD2716, and AZD Racemate. Concentration plotted on log scale. All samples run in duplicate.


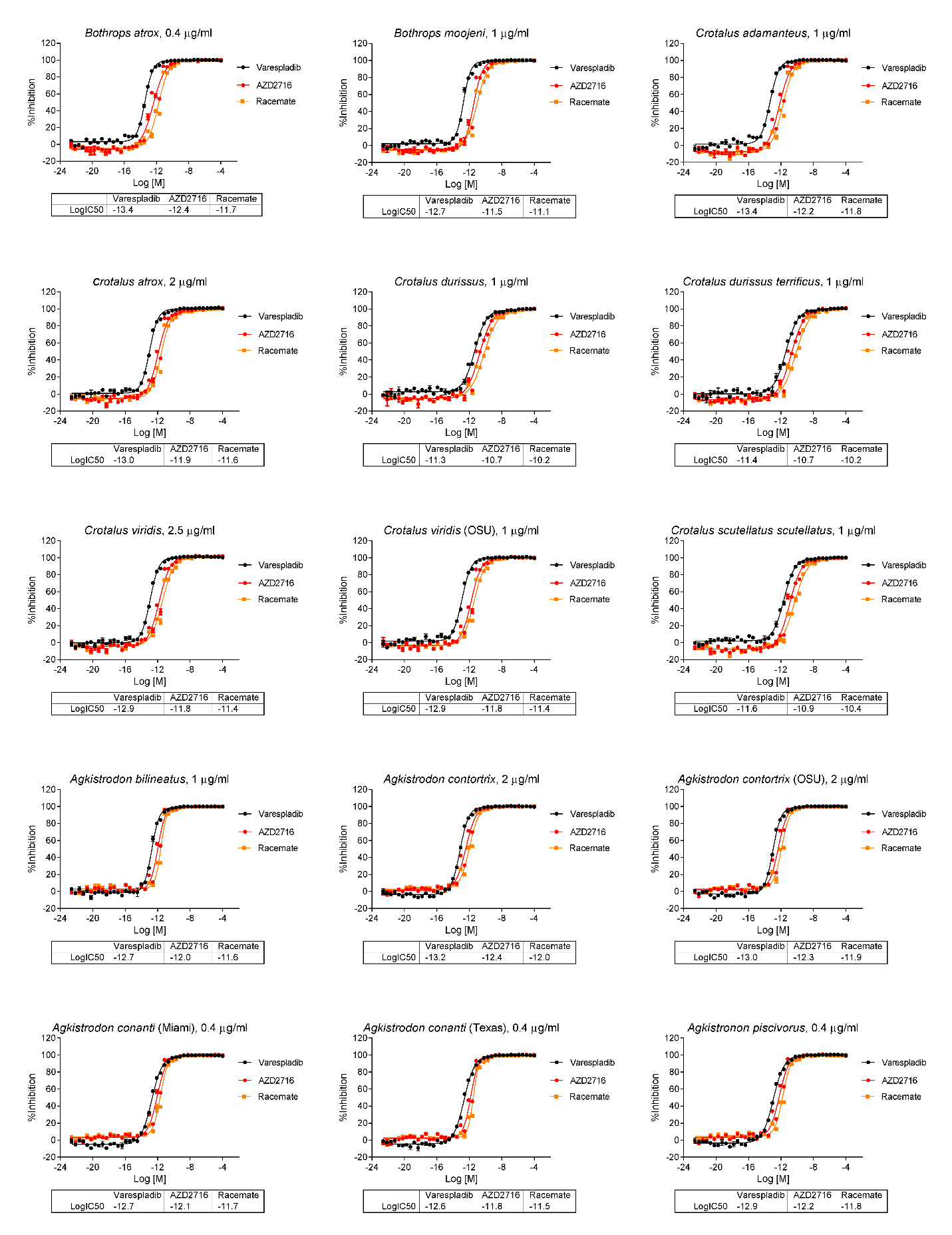


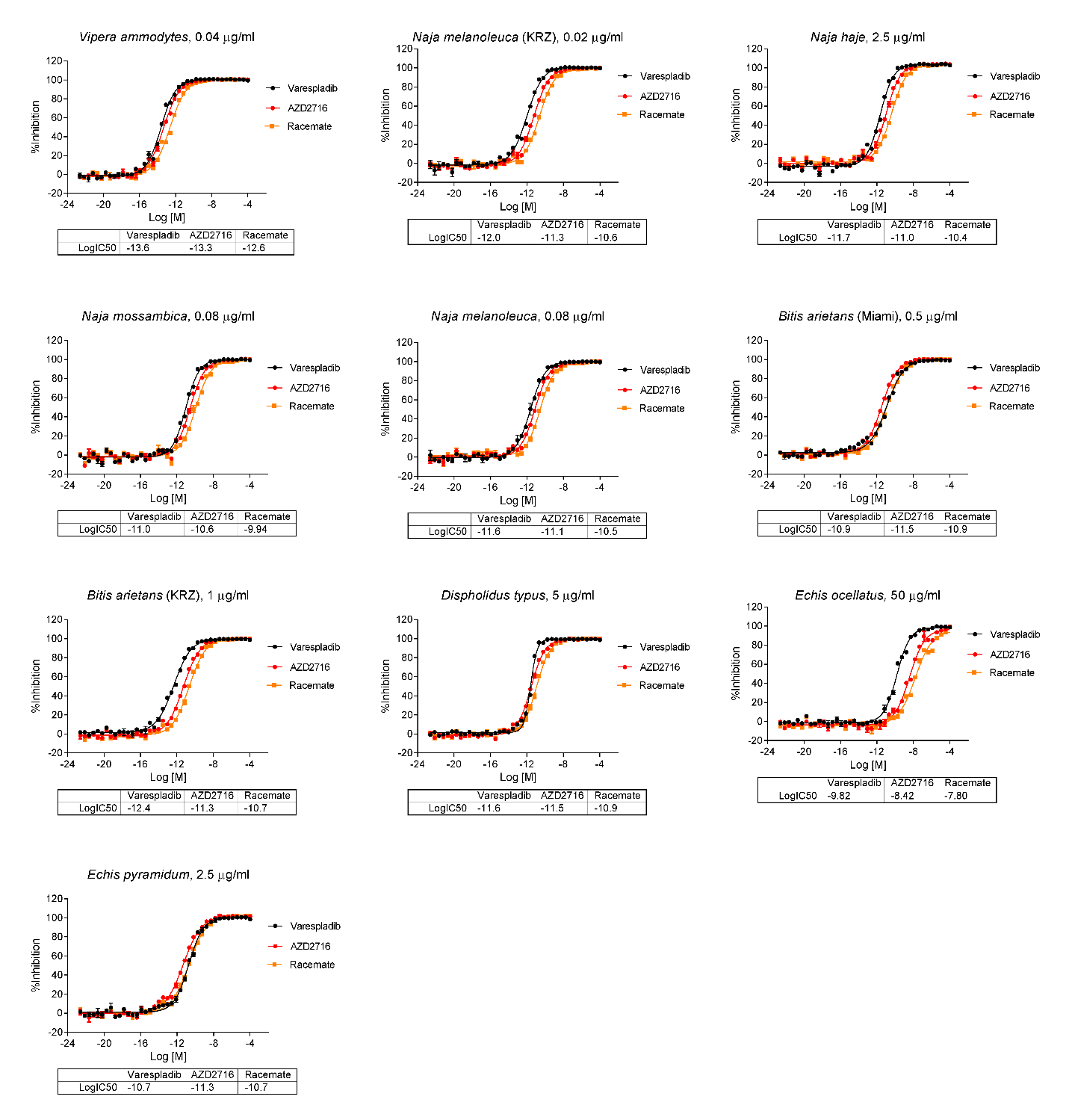


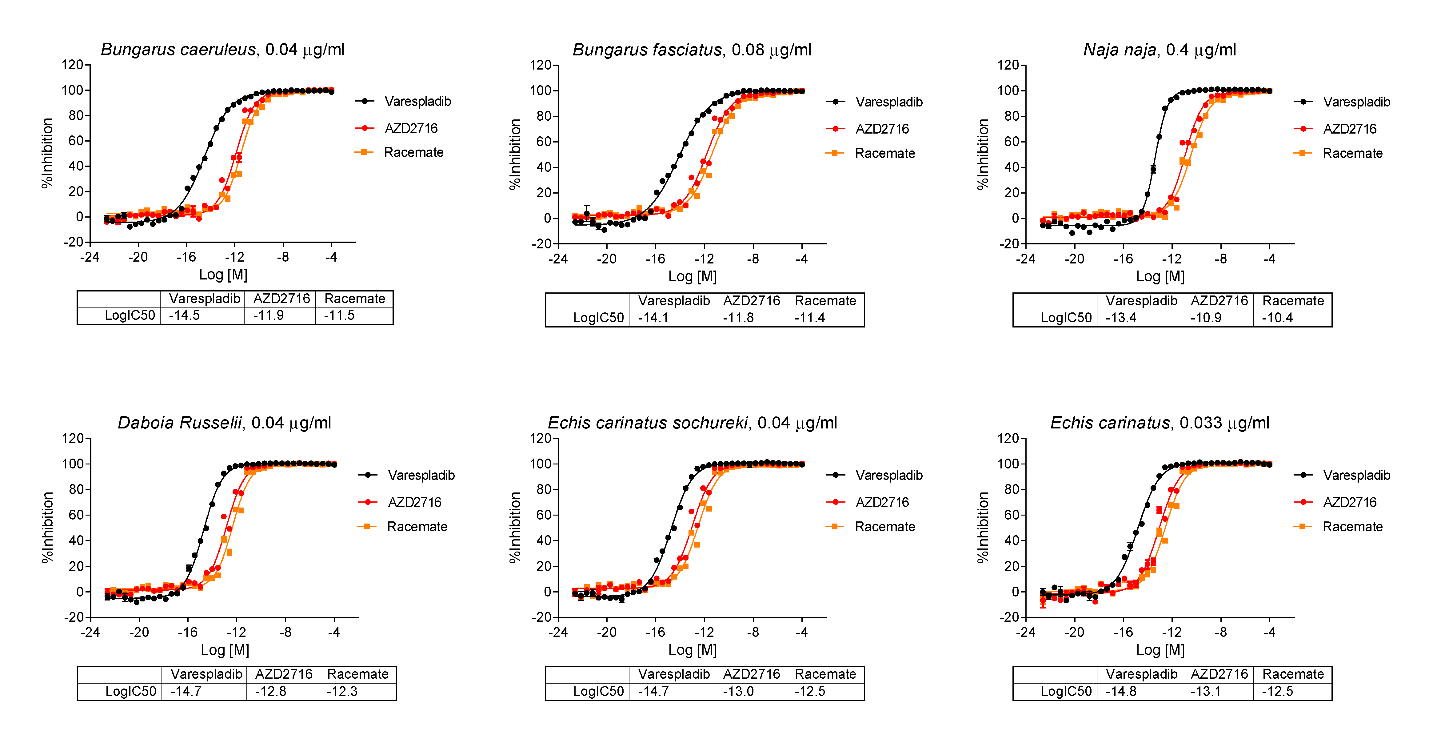


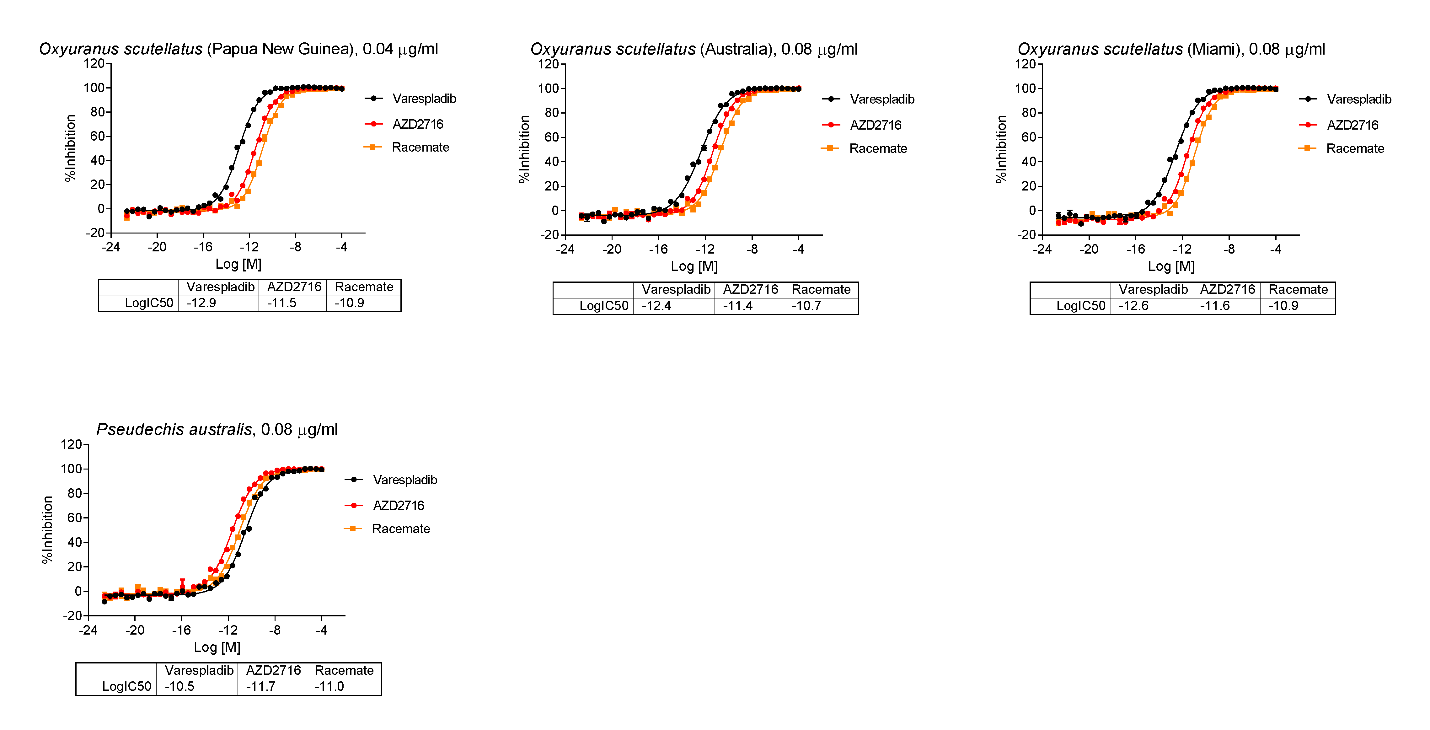


Supplementary Figure 3: Optimization data for *in vitro* studies


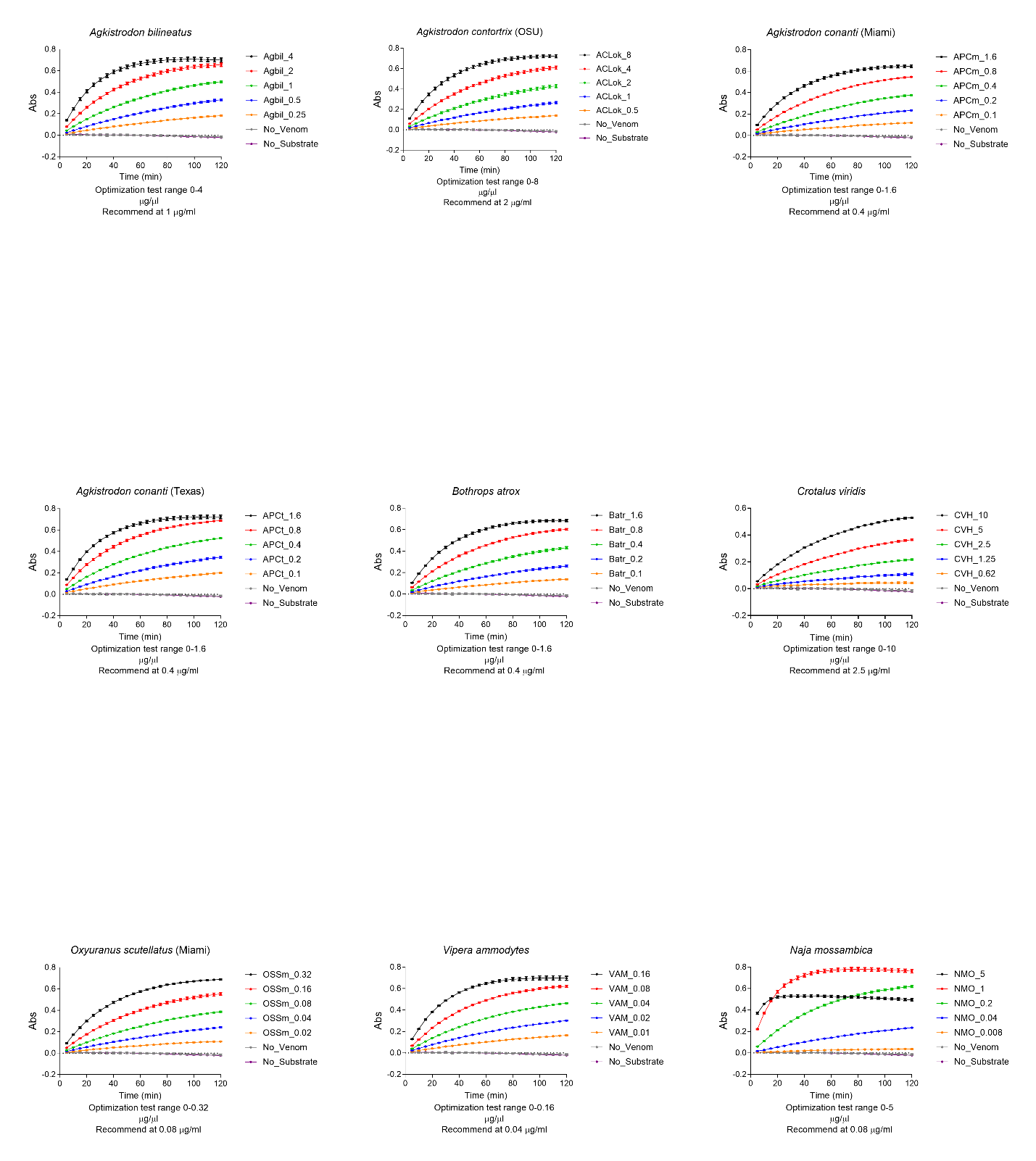


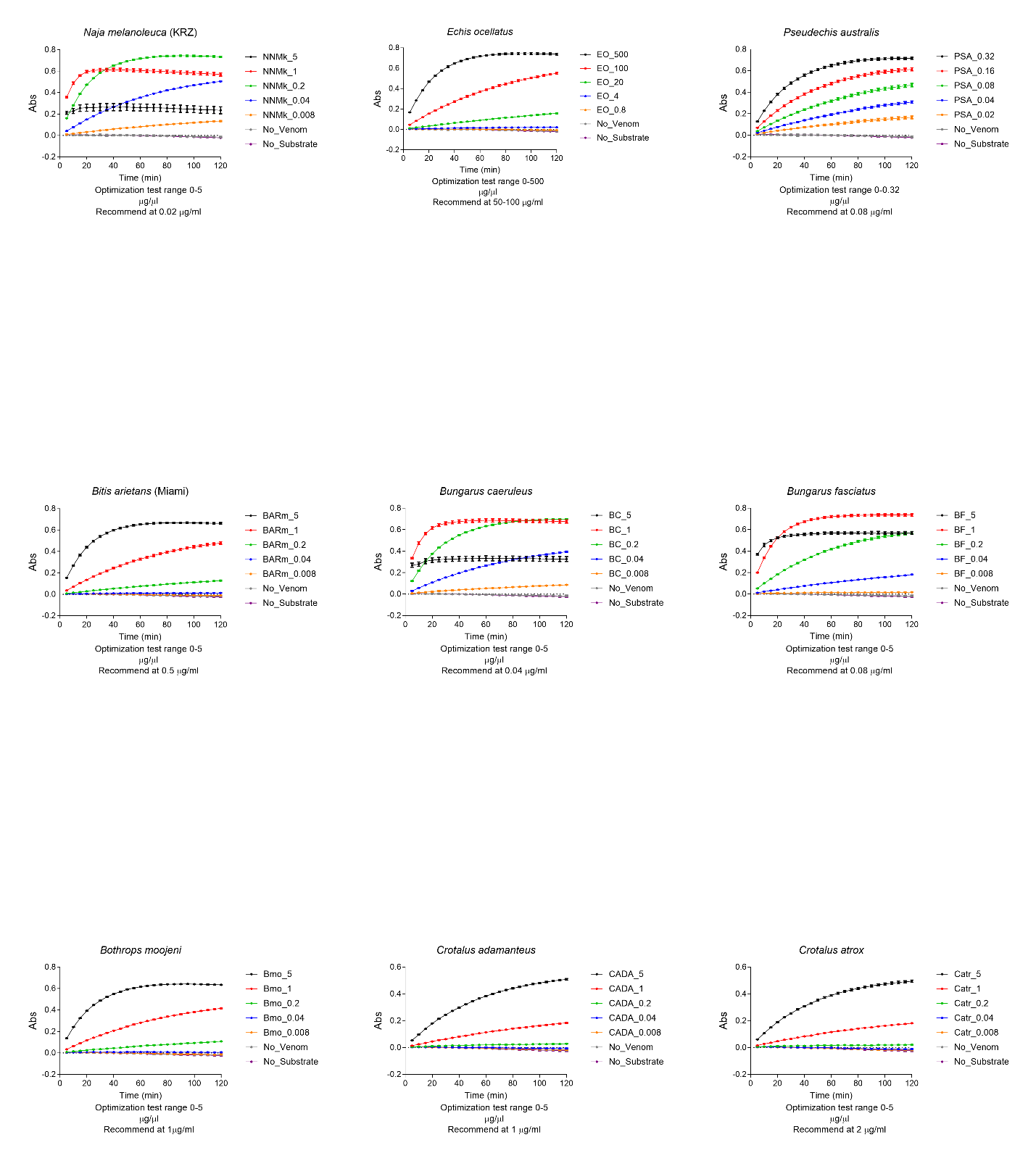


**
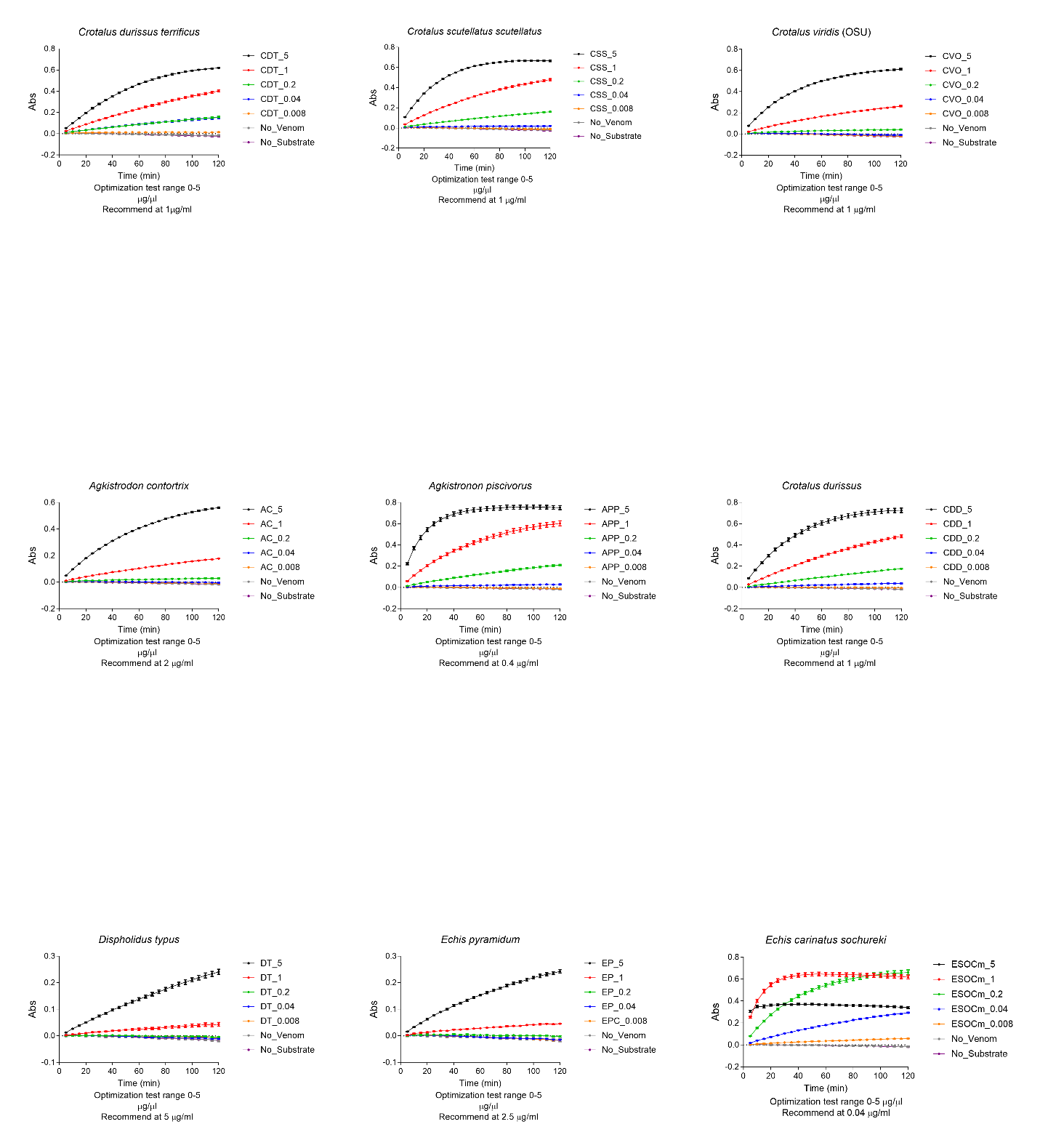
**

**
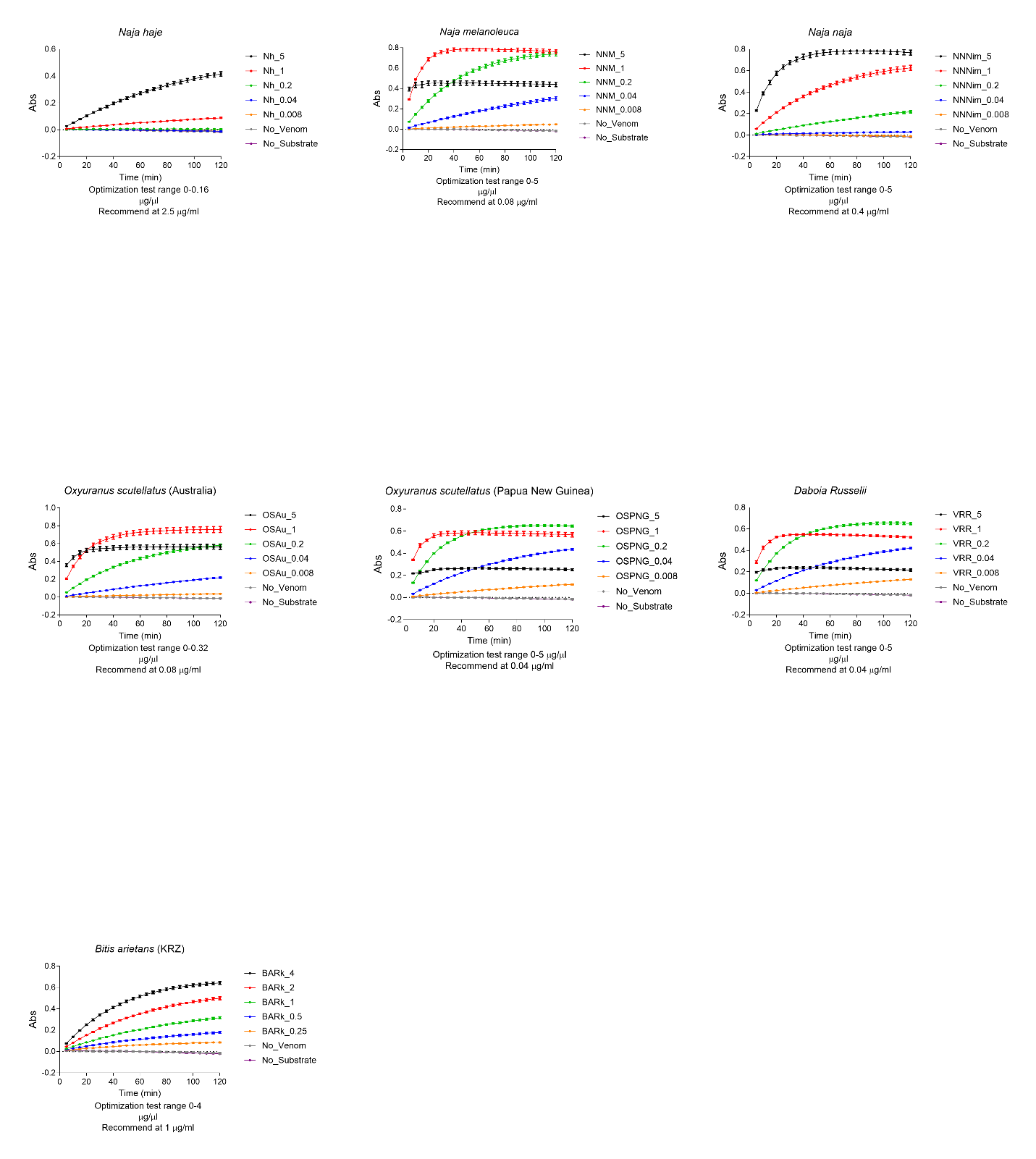
**
